# Genetic diversity analysis in the section *Caulorrhizae* (genus *Arachis*) using microsatellite markers

**DOI:** 10.1590/S1415-47572010005000001

**Published:** 2010-03-01

**Authors:** Darío A. Palmieri, Marcelo D. Bechara, Rogério A. Curi, Jomar P. Monteiro, Sérgio E. S. Valente, Marcos A. Gimenes, Catalina R. Lopes

**Affiliations:** 1 Departamento de Ciências Biológicas, Faculdade de Ciências e Letras de Assis, Universidade Estadual Paulista, UNESP, Assis, SPBrazil; 2 Universidade de Marília, Marília, SPBrazil; 3 Departamento de Melhoramento e Nutrição Animal, Faculdade de Medicina Veterinária e Zootecnia, Universidade Estadual Paulista Júlio de Mesquita Filho', Botucatu, SPBrazil; 4 Department of Medicine, Division of Infectious Diseases and Geographic Medicine, Stanford University, Stanford, CAUSA; 5 Departamento de Biologia, Centro de Ciências da Natureza, Universidade Federal do Piauí, Teresina, PIBrazil; 6 Embrapa Recursos Genéticos e Biotecnologia, Brasília, DFBrazil; 7 Departamento de Genética, Instituto de Biociências, Universidade Estadual Paulista Júlio de Mesquita Filho', Botucatu, SPBrazil

**Keywords:** *Arachis*, genetic diversity, germplasm, microsatellites, molecular markers

## Abstract

Diversity in 26 microsatellite loci from section *Caulorrhizae* germplasm was evaluated by using 33 accessions of *A. pintoi* Krapov. & W.C. Gregory and ten accessions of *Arachis repens* Handro. Twenty loci proved to be polymorphic and a total of 196 alleles were detected with an average of 9.8 alleles per locus. The variability found in those loci was greater than the variability found using morphological characters, seed storage proteins and RAPD markers previously used in this germplasm. The high potential of these markers to detect species-specific alleles and discriminate among accessions was demonstrated. The set of microsatellite primer pairs developed by our group for *A. pintoi* are useful molecular tools for evaluating Section *Caulorrhizae* germplasm, as well as that of species belonging to other *Arachis* sections.

## Introduction

The genus *Arachis* comprises nine taxonomic sections, *viz*., *Arachis*, *Caulorrhizae*, *Erectoides*, *Extranervosae*, *Heteranthae*, *Procumbentes*, *Rhizomatosae*, *Trierectoides* and *Triseminatae*, (Krapovickas and Gregory (1994), and includes both annual and perennial species. In this genus, most secies are acceptable as versatile forage plants. Nevertheless, more recent studies have provided abundant information on the potential and effective commercial use of accessions from the sections *Caulorrhizae* and *Rhizomatosae* (Loch and Ferguson, 1999; Teguia, 2000). Section *Caulorrhizae* is represented by only two stoloniferous species, *Arachis pintoi* Krapov. & Gregory and *Arachis repens* Handro. Both are native of valleys of the rivers Jequitinhonha, Araçuai, São Francisco and Paranã, the latter a tributary of the Tocantins, in Central Brazil.

*Arachis pintoi* is assuming increasing importance in the production of forage in tropical and sub-tropical areas, whereas *A. repens* is used as an ornamental plant, as well as for ground-cover in substitution of several species of common grass. Most of their cultivars were based on the two original accessions, *A. pintoi* GK12787 and *A. repens* GKP10538, which apparently represent extreme morphological types, with the occurrence of intermediate forms (Valls and Simpson, 1994). The basic use of the *A. pintoi* GK 12787 accession has been for developing forage cultivars in Australia, Bolivia, Brazil, Colombia, Costa Rica, Honduras and Venezuela (Valls, 1996).

Lately, the number of accessions available in both species has increased, with the current maintenance of over 150 in the *Arachis* Germplasm Bank (EMBRAPA Recursos Genéticos e Biotecnologia, Brasília, DF, Brazil). Furthermore, a program for agronomic appraisal and production of intra- and inter-specific hybrids from section *Caulorrhizae*, as well as progenies from accessions with high forage potential, has been developed (Carvalho S, PhD Thesis, UNESP, São Paulo, 2000). The significant genetic variability in available germplasm, both in accessions and hybrids, requires conservation, investigation and economical exploitation (Gimenes *et al.*, 2000).

Several genetic markers have been used to estimate the genetic variability in species of section *Caulorrhizae*, including morphological characters (Monçato L, MSc Dissertation, UNESP, São Paulo, 1995), seed storage proteins (Bertozo and Valls, 2001), isozymes (Maass *et al.*, 1993) and RAPDs (Gimenes *et al.*, 2000) These markers were useful for the characterization of genetic variation in both species, but they offered limited informative content since some detected low levels of polymorphism (morphological characters, isozymes and seed proteins). RAPDs, on the other hand, yielded more complex band patterns (RAPDs). Due to their limitations, these markers were incapable of providing relevant information regarding important points for the conservation and use of the species, such as an estimate of the cross-pollination rate, identification of hybrids among species, and accurate estimation of genetic variability.

Microsatellites or simple sequence repeats (SSRs), the most informative molecular markers, have not been extensively used with section *Caulorrhizae* species (Palmieri *et al.*, 2002; 2005). These sequences, besides being abundant and distributed throughout eukaryotic genomes, are highly polymorphic, inherited codominantly and reproducible, with simple screening requirements (Rosseto *et al.*, 2002). The high polymorphism in microsatellite loci is due to DNA polymerase slippage during replication, and (or) unequal crossing-over, thereby resulting in differences in the copy numbers of the core sequences (Schlötterer and Tautz, 1992). Microsatellites have been extensively used in genetic mapping and genome analysis (Brondani *et al.*, 1998; Li *et al.*, 2000), genotype identification, variety protection (Giancola *et al.*, 2002), seed purity evaluation, germplasm characterization (Brown *et al.*, 1996; Hokanson *et al.*, 1998), diversity studies (Métais *et al.*, 2002), marker-assisted breeding (Weissing *et al.*, 1998), and gene and quantitative trait loci analysis (Fahima *et al.*, 1998; Brondani *et al.*, 2002).

From recent studies, 18 microsatellite markers from *A. pintoi* have been described. The utility of these markers in evaluating genetic variability in section *Caulorrhizae* (20 accessions of *A. pintoi* and five of *A. repens*) has been demonstrated (Palmieri *et al.*, 2002, 2005). In the present study, we used 19 previously described microsatellite markers and seven new primer pairs to estimate genetic variation in accessions of *A. pintoi* and *A. repens*.

## Material and Methods

###  Plant material

Thirty-three accessions of *A. pintoi* and ten of *A. repens* were analyzed (Table 1). The samples were obtained from Dr. José F.M. Valls, curator of Wild *Arachis* Germplasm Bank, EMBRAPA Recursos Genéticos e Biotecnologia, Brasília, DF, Brazil, and from Dr. Sandremir de Carvalho, the Fundação Faculdade de Agronomia “Luiz Meneghel”, Bandeirantes, PR, Brazil. In the ArLag (*Arachis sp.*) accession, collected at Botucatu, SP, Brazil, the morphological type appeared to be closer to *A. repens* accessions, although definitive botanical identification was not possible.

###  Source of microsatellites primer pairs

Nineteen primer pairs had already been described by Palmieri *et al.* (2002, 2005) and Hoshino *et al.* (2006), and seven new ones are described herein (Table 2). All the microsatellites used were isolated by applying library-enrichment protocol adapted from Kijas *et al.* (1994). The Primer 3 (Rozen and Skaletsky, 2000) program was employed for designing all the primer pairs, according to the following criteria: Tm of 50 to 60 °C (Tm difference between each primer within a pair was maintained below 3 °C), length of PCR products ranging from 100 to 350 bp and GC-content maintained around 50%. All primer pairs were synthesized by Invitrogen, SP, Brazil. BLAST searches were performed for all microsatellite sequences using blastx program to determine whether the microsatellites were associated with conserved gene regions (Altschul *et al.*, 1997). These searches were based on the full-length sequence from which the primer pairs were designed.

###  DNA extraction

Genomic DNA was extracted using the protocol described by Grattapaglia and Sederoff (1994) with minor modifications as to DNA precipitation. DNA quality was checked with electrophoresis in 1% agarose gels, and concentration estimated by spectrophotometry (Spectronic, Inc., Rochester, NY, USA).

###  DNA amplification and electrophoresis

PCR reactions contained 15 ng of genomic DNA, 1U of *Taq* DNA polymerase (Amersham Biosciences), 1x PCR buffer (200 mM Tris pH 8.4, 500 mM KCl), 1.5-2.0 mM MgCl_2_, 200 μM of each dNTP, and 0.4 μM of each primer, in a final reaction volume of 10 μL. All PCR amplifications were carried out in a PTC100 thermocycler (MJ Research, Inc., Watertown, MA, USA). PCR conditions were 96 °C for 5 min, followed by 32 cycles of 96 °C for 30 s, X ºC for 45 s, 72 °C for 1 min, with a final extension of 10 min at 72 °C. The X value for each primer pair is shown in Table 2. PCR reactions were mixed with equal volumes of loading buffer (95% formamide, 0.01% bromophenol blue, 0.01% xylene cyanol, 0.5% NaOH 0.2 M), denatured at 95 °C for 5 min, cooled on ice and loaded onto the gel. PCR products were separated in denaturing polyacrylamide gels (6% acrylamide/bisacrylamide, 29:1, 5 M urea in TBE, pH 8.3) at 60 W for 4 h in 1x TBE buffer. DNA fragments were visualized by silver staining. The silver staining procedure consisted of 10 min in 10% ethanol/1% acetic acid solution, staining for 15 min in 0.2% (w/v) silver nitrate solution, and rinsing for 30 s in deionized water, and developing in 30 g/L of NaOH/10 mL/L of 37% formaldehyde solution for about 10 min or until bands became visible.

###  Data collection and analysis

Fragment sizes were estimated by comparison with a 10-bp DNA ladder (Life Technologies) using Gene Profiler 4.03 for Windows software, evaluation edition (Scanalytics, Inc., Fairfax, VA, USA). Bands with the same mobility were considered identical. Assuming the absence of null alleles, the presence of only one fragment of a given microsatellite indicated homozygosis. The Ap172 primer pair amplified a putative duplicate locus, and for this reason the amplification of two independent loci for this marker was considered. PopGene software (version 1.31; Yeh *et al.*, 1999) was used to estimate genetic diversity based on the following indexes: polymorphic information content, allele number (observed and effective) per locus, allelic frequencies, observed (*H*_*O*_) and expected (*H*_*E*_) heterozygosities. Allelic polymorphic information content (PIC) was calculated for each microsatellite locus using the formula: 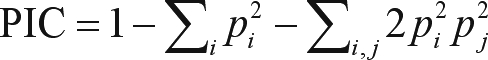, where *p*_*i*_ and *p*_*j*_ are the frequencies of the *i*^th^ and *j*^th^ alleles in the population (Weber, 1990). PIC values provided an estimate of the discriminatory power of a marker by taking into account, not only the number of alleles at a locus, but also their relative frequencies in the population under study. Markers with a large number of alleles occurring at equal frequencies will always have the highest PIC values (Senior *et al.*, 1998). Effective alleles per locus (n_e_) were calculated according to Weir (1989) with the formula 1/(1 - *H*_*E*_). *H*_*E*_*,* the expected heterozygosity per locus, is equal to 
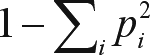
, where *p*_*i*_ is the frequency of the *i*^th^ allele at the locus. The Unweighted Pair-Group Method was applied for cluster analysis, using Arithmetic Averages (UPGMA) based on unbiased genetic distance measures (Nei, 1978).

## Results and Discussion

Twenty six microsatellite primer pairs were tested. Nineteen pairs (73%; Ap18, Ap22, Ap23, Ap33, Ap40, Ap45, Ap48, Ap152, Ap154, Ap158, Ap161, Ap166, Ap172, Ap175, Ap176, Ap183, Ap187, Ap190 and Ap196) allowed the detection of polymorphism while seven did not (27%; Ap10, Ap32, Ap35, Ap38, Ap46, Ap164 and Ap177) when all samples of the two species were considered. Sequences of Ap10, Ap18, Ap35, Ap45, Ap164, Ap177 and Ap190 are being presented for the first time. Locus Ap45 was mono-morphic only in *A. pintoi,*whereas Ap48 was monomorphic only in *A. repens* accessions (Table 2). Polymorphism in Ap40 (17 repeats) and Ap176 (18 repeats) had already been revealed in previous studies on *Arachis* genetic variability (Bravo *et al.*, 2006; Hoshino *et al.*, 2006; Angelici *et al.*, 2008), as well as in the present study.

The number of monomorphic loci was high by accounting that each primer pair that did not allow detection of polymorphism was adjacent to regions containing a high number of repeats, these ranging from 19 (Ap32) to 58 (Ap35) repeats. Among the ones that did not detect any polymorphism four are described in this paper and two (Ap32 and Ap38) were previously used in three studies on genetic variability in *Arachis* (Bravo *et al.*, 2006; Hoshino *et al.*, 2006; Angelici *et al.*, 2008), all with similar results. We tested the latter two primer pairs because Hoshino *et al.* (2006) studied only one accession of each species of section *Caulorrhizae*, whereas Bravo *et al.* (2006) and Angelici *et al.* (2008) used these two primers in other sections of genus *Arachis*. Thus, we expected additional information from these primers by using samples of the species from which they had been isolated. It may be that the areas targeted by the two primer pairs are within conserved regions of the genome. There was no similarity between the sequences used to design primers for these six microsatellites and any nucleotide or protein sequence in GenBank.

The Ap172 primer pair amplified a putative duplicated locus. At first, the double-band pattern was interpreted as a technical artifact, but after several attempts to optimize the amplification reaction, the band pattern still remained, thereby implying locus duplication. Amplification of duplicated loci has been observed in several species, such as *Glycine max* (L.) Merr. (Powell *et al.*, 1996; Peakall *et al.*, 1998), *Zea mays* L. (Senior *et al.*, 1998), *Vigna radiata* (Kumar *et al.* 2002) and *Cicer arietinum* L. (Sethy *et al.*, 2003). In rice and sunflowers, the amplification of double-band patterns has also been attributed to the occurrence of a duplication process within the genome itself, as well as to the evolution of families of repetitive sequences (Akagi *et al.*, 1998; Paniego *et al.*, 2002). In the amphidiploid *A. hypogaea*, amplification of duplicated loci was reported by Hopkins *et al.* (1999), and duplication at several genomic regions by Burow *et al.* (2001). Despite *A. pintoi* and *A. repens* being diploid species, gene duplication is not rare in the genus *Arachis*, and it could have happened to Ap172.

In this study, only Ap172 and Ap176 sequences showed similarity at the amino acid level to seryl-tRNA synthetase (57% identity, 76% similarity) and lipoxygenase (41% identity, 47% similarity) of plants, respectively. These stretches of similarity are localized adjacent to microsatellite sequences (data not shown). A like occurrence was reported by Peakall *et al.* (1998) in soybean. These authors found a similarity of 96% at the amino acid level between a microsatellite sequence and a seryl-tRNA synthetase of *Arabidopsis thaliana*. These data seem to be in agreement with observations from several authors (Tóth *et al.*, 2000; Li *et al.*, 2002; Morgante *et al.*, 2002), in the sense that microsatellite sequences are present both in coding and non-coding regions of nuclear and organellar genomes.

A total of 196 putative alleles were detected at 20 polymorphic loci. It was assumed that fragments of different lengths were different alleles. The number of alleles ranged from two at Ap45 to 23 at Ap18 (a mean of 9.8 alleles/locus) (Table 3). The effective number of alleles ranged from 1.07 at Ap45 to 16.7 at Ap18 (Table 4). In *A. pintoi*, 174 alleles were detected distributed among the 19 polymorphic loci (mean of 9.2 alleles/locus), their fragment sizes ranging from 140 bp (Ap161) to 306 bp (Ap152). In *A. repens* accessions, 99 alleles, with fragment sizes ranging from 140 bp (Ap161) to 304 bp (Ap33), were detected among 19 polymorphic loci (mean 5,2 alleles/locus) (Table 3). Ninety-nine alleles (49%) were exclusively present in *A. pintoi* and twenty-one alleles (10.7%) were found in *A. repens* accessions only. Seventy-ninealleles (40.3%) were shared between the two species (data not shown). On using RAPDs, Gimenes *et al.* (2000) obtained lower values for exclusive fragments for these two species (22% in *A. pintoi* and 5% in *A. repens*) and a higher value for shared fragments (73%). Based on these results, they discussed the difficulty in justifying the separation into two species. Our data could reinforce a separation of these species into two taxa, as the higher values observed were due to the codominance and informativeness of microsatellite markers, thereby allowing us to distinguish and better estimate the genetic diversity within the analyzed germplasm.

Data on allelic polymorphic information content (PIC), and observed (*H*_*O*_) and expected (*H*_*E*_) heterozygosities per locus are presented in Table 4. PIC values ranged from 0.0651 at Ap45 to 0.9369 at Ap18, with an average value of 0.6423 when considering 20 polymorphic loci (Table 4). Average observed heterozygosities at 20 loci for the whole *A. pintoi* and *A. repens* sample were 0.5788, 0.5820 and 0.5861, respectively (Table 4), and average expected heterozigosities for the whole sample, *A. pintoi* and *A. repens* accessions were 0.6753, 0.6553 and 0.6202, respectively (Table 4). Mean values of observed heterozygosity (*H*_*O*_) were lower than the *H*_*E*_ values estimated from allele frequencies. At some loci, *H*_*O*_ values were higher than *H*_*E*_ (Ap22, Ap23, Ap154, Ap172a, Ap172b, Ap187, and Ap190). The variability observed in *A. pintoi* could be the consequence of crosses between different accessions that had been vegetatively maintained at experimental plots. Thus, the high observed heterozygosity at some loci could be attributed to the presence of parentals carrying different alleles, thereafter being sustained through the vegetative propagation methods used in conserving accessions.

**Figure 1:**
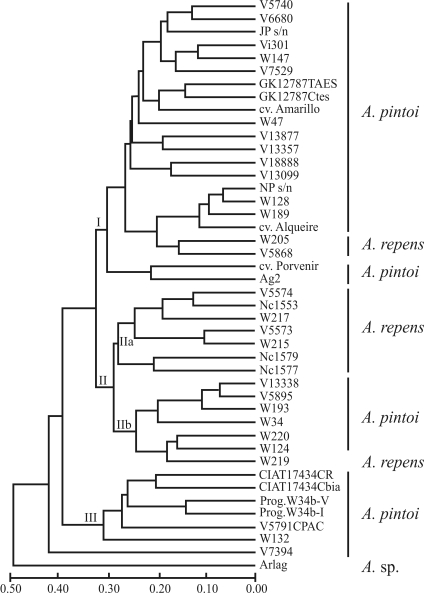
UPGMA dendrogram of 33 accessions of *A. pintoi* and ten of *A. repens*. The distance matrix was estimated by the Nei (1978) coefficient using 27 microsatellite loci. Clades were defined by roman numerals at the nodes. Individual accessions and species are listed to the right of the dendrogram.

The dendrogram showing the relationships among *A. pintoi* and *A. repens* accessions is presented in Figure 1. Cluster analysis allowed the discrimination of all individuals from the two species. Such differentiation was also obtained using RAPD markers (Gimenes *et al.*, 2000). However, microsatellites should be the marker of choice because they are much more effective and have higher reproducibility since longer primer pairs are used instead of unique short primers that allows multiple loci amplification, which makes the analysis difficult.

Three major groups (I, II and III) were formed in the tree. In general, *A. pintoi* accessions were positioned in all the three major groups, with a mean genetic distance among them of 0.295, ranging from 0.064 (between NP s/nº and WPn 128) to 0.566 (between W 34 and CIAT 17434 – Maní Mejorador). Group I was formed by 20 *A. pintoi* accessions and only two *A. repens* (WPn 205 and V 5868). Two subgroups were observed in Group II. Subgroup IIa was formed by seven out of ten *A. repens* accessions with a mean genetic distance of 0.232. Six of these were collected in Minas Gerais State, Brazil. Subgroup IIb was represented by six *A. pintoi* accessions (VPzAg 13338, VW 5895, WPn 193, W 34, WPn 220 and WPn 124) and only one *A. repens* (WPn 219). Group III was formed solely by *A. pintoi* accessions.

The longest genetic distance (0.582) was obtained between the accessions CIAT 17434 – Maní Mejorador (*A. pintoi*) and WPn 215 (*A. repens*), whereas the shortest (0.064) was between two *A. pintoi* accessions (NP s/nº and WPn 128). The VSa 7394 (*A. pintoi*) accession, the most diverse, was positioned outside the three major groups (Figure 1). Tree analysis showed that the species could not be characterized based on polymorphism detected by using 20 microsatellite loci, since accessions of each species were not entirely grouped together. Likewise, Bravo *et al.* (2006) and Hoshino *et al.* (2006) did not resort to microsatellites when characterizing *Arachis* species. They pointed out that this was probably due to: 1 – high microsatellite-detected polymorphism, requiring larger samples for adequate representation of species variability; and 2 – the existence of homoplasies (fragments of the same size but from different loci that have no common origin). These same factors could possibly have affected the results obtained in this study. However, we believe the main reason is that crossability in *A. pintoi* and *A. repens* is high (86.7%, Krapovickas and Gregory, 1994), these being considered by some authors as a single species (Gimenes *et al.*, 2000). As mentioned above, differentiation between *A. repens* and *A. pintoi*, as observed in the present study, was greater than that observed by Gimenes *et al.* (2000). We consider this to be a relevant result, because it shows that the primary gene pool of these species probably has a wider base than was detected by the RAPD data.

It has been demonstrated that the set of microsatellite markers previously described and used here provides a powerful tool for germplasm characterization analysis of *A. pintoi* and *A. repens* species. Among the primer pairs presented in this study, 21 are readily available. These primers could be useful in all the steps from conservation to the use of germplasm. The existence of duplicates, mislabeling and loss of integrity due to physical contamination, cross-pollination or genetic drift are realities, so these markers could be used as an aid in evaluating these events in the germplasm collection. Furthermore, they could also be used in identifying accessions and cultivars and for selecting parents for hybridization.

## Figures and Tables

**Table 1:** Germplasm of section *Caulorrhizae* analyzed in this study.

Samples	Code	Collector's number ^a^	Origin	River basin ^b^
*A. repens*	012114	V 5868	São Gabriel-RS	-
	014770	VSW 6673	Várzea da Palma-MG	SF
	014788	VSW 6674	Várzea da Palma-MG	SF
	029190	Nc 1563	Buenópolis-MG	SF
	029203	Nc 1577	Vitória-ES	SF
	029220	Nc 1579	Januaria-MG	SF
	032310	WPn 205	Pres. de Moraes-MG	SF
	032352	WPn 215	Buenópolis-MG	SF
	032379	WPn 217	Buenópolis-MG	SF
	032395	WPn 219	Bocaiúva-MG	JQ
*A. pintoi*	012122	VW 5895	Unaí-MG	SF
	014982	VSW 6740	Pres. Juscelino-MG	SF
	015083	VSW 6784	Sta Maria da Vitória-BA	SF
	015121	V6791-CPAC	Faz. Genipapo-GO	PR
	015253	W 34	Fco. Badaró-MG	JQ
	015598	W 47	Brasília-DF	-
	016357	Vi 301	Araçuaí-MG	JQ
	016683	VSa 7394	Brasília-DF	-
	020401	VRVe 7529	Campinas-SP	-
	030261	VFaPzSv 13099	Araçuaí-MG	JQ
	031305	WPn 124	Buritis-MG	SF
	031321	WPn 128	Buritis-MG	SF
	031364	WPn 132	Unaí-MG	SF
	031461	WPn 147	Jaíba-MG	SF
	031534	VPzBmVaDb 13357	Jussari-BA	JQ
	032191	WPn 189	F.da Mata-BA	SF
	032239	WPn 193	Sta Maria da Vitória-BA	SF
	032409	WPn 220	Eng. Navarro-MG	SF
	034100	VPzAg 13338	Formosa-GO	PR
	034347	VApW 13877	Formosa-GO	PR
	034355	VApW 13888	Buritonópolis-GO	PR
	N.D.	Prog. W34b – I	N.A.	-
	N.D.	Prog. W34b – V	N.A.	-
	012122	CIAT 18744 - cv. Porvenir	Unaí-MG	JQ
	013251	GK 12787 - Ctes	Argentina	JQ
	013251	GK 12787 - TAES	U.S.A.	JQ
	013251	CIAT 17434 - Maní Forrajero Perenne	Colombia	JQ
	013251	CIAT 17434 - Maní Mejorador	Costa Rica	JQ
	013251	GK 12787 - cv. Amarillo	Australia	JQ
	037036	NP s/nº	Rio Pardo-RS	-
	037036	cv. Alqueire	Rio Pardo-RS	-
	031828	JP s/nº - cv. Belmonte	Itabuna-BA	JQ
	031895	Ag2 (2n = 30)	San José-CRA	-
*A. sp.*	N.D.	ArLag	Botucatu-SP	-

^a^Collectors – Ap = A. Peñaloza, Bm = B. Maass, Db = M. Bechara, Fa = L. Faraco, Nc = N. Costa, NP = N. Perez, Pn = P. Pinheiro, Pz = E. Pizarro, R = V. Rao, S = C. Simpson, Sa = J. Santos, Sv = Silva, Ve = R. Veiga, Vi = J. Vieira, V = J. Valls, Va = S. Valente, W = W. Werneck. ^b^River basin – JQ = Jequitinhonha, PR = Paranã, SF = São Francisco.

**Table 2:** Primer sequences, characteristics and source of the 26 microsatellite loci used in estimating genetic variation in germplasm of section *Caulorrhizae*.

Locus	Primer Sequences (5' to 3')	Repeat motif	Annealing temp. (ºC)	Size (bp)^a^	Accession number	Source of primers
Ap10	GAGGGAGTGAGGGGTTTAG	(AG)_42_	52	144	AY540972	This work
	ATCCCCACCCCTTCTTT					
Ap18	TGCAGCCCACTGTATATTCG	(TA)_36_	52	200	AY540973	This work
	TACACAGCGTAACAACTTATTTAGTG					
Ap32	ATAGGGAGAAGGCAGGGAGA	(TC)_19_	55	148	AY540976	Hoshino *et al.* (2006)
	GATCATGCTCATCATCAACACC					
Ap35	TTAGACTACCAATCTATACGTACA	(GA)_58_	52	202	AY540978	This work
	TCACCGATCCACTTTAAAGACA					
Ap38	GCGAACAAAGGAGGAAGAGA	(CT)_25_	55	154	AY540979	Hoshino *et al.* (2006)
	GCTGGAAGACGTCATGGTTT					
Ap45	TGTGCACACTCAGACTCAACA	(TC)_40_	55	185	AY540980	This work
	TTTAGCCTAGAGCCGAATTCAC					
Ap164	TGGTGGAATTGCAGAGAAC	(AG)_33_	55	213	AY540985	This work
	GATTCAGGCTGCAGATGGAC					
Ap177	CCGAATTCACCGATCCACT	(CT)_35_	55	143	AY540987	This work
	GGGCGATACTGAGCAACGTA					
Ap190	CTGTTTGATCGCCGCTATG	(TC)_17_	55	178	AY540990	This work
	GTCAAGTGCTTCCTCCGATG					
Ap40	CTGTTTGATCGCCGCTATG	(TC)_17_	55	178	AF504067	Palmieri *et al.* (2002)
	GTCAAGTGCTTCCTCCGATG					
Ap46	GAAATCACCGATCCCACTTT	(AG)_22_	55	158	AF504068	Palmieri *et al.* (2002)
	CCATGATTTCATTCGCAAAC					
Ap152	AGAGGATGCAGCGGAGTAGA	(TC)_24_	50	277	AF504069	Palmieri *et al.* (2002)
	CTGGCCAATTCCTATGATCG					
Ap166	CGGCAGTCAACGAAGCTAT	(CT)_14_	50	200	AF504070	Palmieri *et al.* (2002)
	TCGCCAAAGGTTAGATTGC					
Ap175	CCAATAGGCTAATTCAGAAGG	(AG)_22_	50	177	AF504071	Palmieri *et al.* (2002)
	GCCTTATTTTGCGACTGAGG					
Ap176	CCAACACAGGGCTTACCAAG	(AG)_18_	50	222	AF504072	Palmieri *et al.* (2002)
	TCACCGATCCCACTTTTCC					
Ap22	ACTGCACGTCCTCTCTCCTC	(AG)_14_..(GGA)_4_..(GA)_9_	55	255	AY540974	Palmieri *et al.* (2005)
	TGCATCTTCACCAGCCTACA					
Ap23	TGCTCCCAACTGCTACCAA	(AG)_22_	52	199	AY540975	Palmieri *et al.* (2005)
	TGAGCAAGAAGAACGAACGA					
Ap33	CAGCCTAGAGCCGAAAACAC	(CT)_36_	55	161	AY540977	Palmieri *et al.* (2005)
	GATGGCATGGCTGTCAGTAA					
Ap48	ACCGATCCCACTTTTCCAC	(AG)_18_	52	205	AY540981	Palmieri *et al.* (2005)
	CCAAGAATGGCGATTGATTC					
Ap154	TGTCCAAATCACCTGAGACG	(CT)_18_	55	187	AY540982	Palmieri *et al.* (2005)
	GGAACGGAGATGACAGAAGG					
Ap158	GTCTGCAGAGGAGCCAACAT	(AG)_29_	55	115	AY540983	Palmieri *et al.* (2005)
	TCTTCCTCTCCTCGCGTTC					
Ap161	ACCGTCCTCTTCCTCTCCTC	(GT)_32_	55	215	AY540984	Palmieri *et al.* (2005)
	CCCTCTCCAAATGGACACAT					
Ap172	TGCATCTTCACCAGCCTACA	(AG)_14_	55	255	AY540986	Palmieri *et al.* (2005)
	ACTGCACGTCCTCTCTCCTC					
Ap183	CATCGTGTGGAGACGAAGGT	(GA)_23_	55	198	AY540988	Palmieri *et al.* (2005)
	GAACCAACAGAGAGCGGATG					
Ap187	TTCGTCATCGTCGTCGTTC	(AG)_24_	55	179	AY540989	Palmieri *et al.* (2005)
	GTGGTGATGATGACGCAGAA					
Ap196	CGCAAGCTCCTTCTTTCTTG	(AG)_22_	55	197	AY540991	Palmieri *et al.* (2005)
	GCGACGTAAGAAGCTCCAAC					

^a^Determined from cloned sequence.

**Table 3:** E xpected size (bp) and total number of alleles of the 26 microsatellite loci in the section *Caulorrhizae*. The size-range and number of alleles from *A. pintoi* and *A. repens* accessions are presented. Numbers between parentheses represent mean numbers of alleles/locus.

Locus name	Length (bp)	Total alleles	A. pintoi			*A. repens*	
			Size range	No. alleles		Size range	No. alleles
Ap10	114	1	114	1		114	1
Ap18	160-234	23	160-234	20		166-234	11
Ap22	168-178	3	174-178	3		168-178	3
Ap23	228-240	6	228-240	5		232-236	4
Ap32	150	1	150	1		150	1
Ap33	296-304	4	296-300	3		298-304	3
Ap35	192	1	192	1		192	1
Ap38	152	1	152	1		152	1
Ap40	156-192	7	156-192	6		168-188	3
Ap45	180-184	2	180	1		180-184	2
Ap46	148	1	148	1		148	1
Ap48	186-190	3	186-190	3		186	1
Ap152	262-306	14	268-306	10		278-302	7
Ap154	166-176	5	166-176	5		166-172	5
Ap158	206-224	5	296-224	4		210-216	5
Ap161	140-180	12	140-180	10		140-180	5
Ap164	206	1	206	1		206	1
Ap166	160-232	22	160-218	22		166-208	5
Ap172a	242-252	4	244-252	4		242-252	2
Ap172b	174-180	3	174-180	2		174-178	3
Ap175	160-206	15	160-204	15		176-206	5
Ap176	202-264	15	202-264	11		212-224	9
Ap177	138	1	138	1		138	1
Ap183	190-228	16	190-228	16		192-210	8
Ap187	152-194	18	152-192	17		156-194	7
Ap190	152-182	15	152-182	14		158-172	9
Ap196	186-194	4	186-194	4		186-192	3
Total	114-306	203 (7.5)	114-306	182 (6.7)		114-304	107 (4.0)
Polymorphic loci	140-306	196 (9.8)	140-306	174 (9.2)		140-304	99 (5.2)

**Table 4:** Characterization of the 20 polymorphic microsatellite loci in the section *Caulorrhizae*. Polymorphic information content (PIC), effective number of alleles, and observed (*H*_*O*_) and expected (*H*_*E*_) heterozygosities obtained per locus.

Locus	PIC	Overall sample				*A. pintoi*			*A. repens*	
		n_e_^1^	*H*_*0*_	*H*_*E*_ *		*H*_*0*_	*H*_*E*_ *		*H*_*0*_	*H*_*E*_ *
Ap18	0.9369	16.7	0.8077	0.9401		0.9444	0.9383		0.5714	0.8571
Ap22	0.4076	2.09	0.9767	0.5214		0.9688	0.5142		1.0000	0.5450
Ap23	0.7223	4.17	1.0000	0.7604		1.0000	0.7812		1.0000	0.5938
Ap33	0.4476	1.93	0.0227	0.4832		0.0303	0.4844		0.0000	0.4600
Ap40	0.7432	4.45	0.3077	0.7751		0.3000	0.7750		0.0000	0.5000
Ap45	0.0651	1.07	0.0233	0.0673		-	-		0.1000	0.0950
Ap48	0.1624	1.21	0.0270	0.1735		0.0385	0.2374		-	-
Ap152	0.8682	8.18	0.8667	0.8778		0.8000	0.8750		1.0000	0.7800
Ap154	0.6594	3.43	0.9667	0.7083		0.9565	0.6720		1.0000	0.7361
Ap158	0.4646	1.98	0.1724	0.4941		0.1739	0.3677		0.2000	0.7800
Ap161	0.8358	6.63	0.1923	0.8491		0.2000	0.8350		0.2000	0.6600
Ap166	0.9188	13.1	0.5714	0.9235		0.5909	0.9308		0.4000	0.3400
Ap172a	0.4086	2.09	1.0000	0.5227		1.0000	0.5303		1.0000	0.5000
Ap172b	0.4097	2.10	0.9756	0.5235		1.0000	0.5000		1.0000	0.5000
Ap175	0.8465	7.07	0.3448	0.8585		0.3913	0.8251		0.2000	0.5800
Ap176	0.8896	9.80	0.6667	0.8980		0.6250	0.8711		0.7500	0.8438
Ap183	0.8632	7.93	0.6897	0.8740		0.6667	0.8526		0.7143	0.7959
Ap187	0.9170	12.8	0.9643	0.9222		0.9545	0.9215		1.0000	0.8000
Ap190	0.8692	8.36	1.0000	0.8803		1.0000	0.8769		1.0000	0.8250
Ap196	0.4103	1.83	0.0000	0.4537		0.0000	0.3182		0.0000	0.5926
Mean	0.6423	4.59	0.5788	0.6753		0.5820	0.6553		0.5861	0.6202
St.Dev.			0.3975	0.2572		0.3988	0.2705		0.4192	0.1985

^1^Effective number of alleles (Kimura and Crow, 1964). *Nei (1973) expected heterozygosity.
